# Left jackknife position: a novel position for laparoscopic hepatectomy

**DOI:** 10.1186/s40880-017-0190-y

**Published:** 2017-03-20

**Authors:** Jian-Cong Chen, Rong-Xin Zhang, Min-Shan Chen, Li Xu, Jin-Bin Chen, Ke-Li Yang, Yao-Jun Zhang, Zhong-Guo Zhou

**Affiliations:** 1 State Key Laboratory of Oncology in South China, Collaborative Innovation Center for Cancer Medicine, Sun Yat-sen University Cancer Center, Guangzhou, 510060 Guangdong P. R. China; 2 Department of Hepatobiliary Oncology, Sun Yat-sen University Cancer Center, 651 Dongfeng Road East, Guangzhou, 510060 Guangdong P. R. China; 3 Department of Colorectal Cancer, Sun Yat-sen University Cancer Center, Guangzhou, 510060 Guangdong P. R. China

**Keywords:** Laparoscopic hepatectomy, Hepatocellular carcinoma, Segment VI, VII, or VIII, Left jackknife position

## Abstract

**Background:**

Laparoscopic hepatectomy for hepatocellular carcinoma (HCC) located in segment VI, VII, or VIII of the liver is usually difficult because of poor operative exposure, due to the unique anatomical structure. In this study, we evaluated the practice of laparoscopic hepatectomy with the left jackknife position for patients with HCC located in segment VI, VII, or VIII.

**Methods:**

A total of 10 patients were enrolled to undergo laparoscopic hepatectomy with the left jackknife position. Tumors located in segment VI, VII, or VIII were assessed by preoperative dynamic computed tomography or magnetic resonance imaging. Operation time, intraoperative blood loss, postoperative fasting time, postoperative drainage time, major postoperative complications, and duration of postoperative hospital stay were recorded.

**Results:**

All surgeries were successfully completed. None of the patients required conversion to open surgery during the procedure, and no serious postoperative complications were observed. The median tumor size was 31 mm (range 23–41 mm) in diameter, the mean operation time was 166 ± 38 min, the mean intraoperative blood loss was 220 ± 135 mL, and the median postoperative hospital stay was 4 days (range 2–7 days).

**Conclusions:**

For HCC located in segment VI, VII, or VIII, laparoscopic hepatectomy with this novel position—the left jackknife position—is safe and effective during tumor resection by exposing a sufficient operating field.

*Trial registration* ClinicalTrials.gov ID: NCT02809287

## Background

Since the first report of laparoscopic resection of a benign hepatic tumor by Reith in 1991 [1], the laparoscopy has been widely used in the treatment of liver diseases [2, 3]. In recent years, with the continuous improvement of laparoscopic techniques and equipments, laparoscopic hepatectomy has been more frequently used [4]. The number of reported cases of laparoscopic hepatectomy has steadily increased, especially the marked increase of major and anatomic resections since 2009 although local resections still comprise the vast majority of procedures in clinical practice [5]. Laparoscopic hepatectomy has also been used for liver graft procurement for organ transplantation because of its advantages, such as minimal incision and quick postoperative recovery [6–8]. Based on its superiority in amplifying operative vision for deep anatomic sites and a wide operative area, laparoscopic hepatectomy has been well recognized globally [6, 9, 10]. Generally speaking, for lesions located in the left, front, or lower part of the liver, corresponding to Couinaud segment II, III, IVb, V, or VI, a laparoscopic hepatectomy is recommended [11, 12]; however, for lesions located in segment VII or VIII, the surgery is technically difficult due to poor exposure of the operating field [13]. Therefore, we employed the left jackknife position in laparoscopic hepatectomy to better expose lesions in segment VI, VII, or VIII, aiming to shorten operation time and reduce bleeding.

## Patients and methods

### Patient enrollment

This prospective study was approved by the Institutional Review Board at Sun Yat-sen University Cancer Center, in Guangzhou, China, and carried out in accordance with approved guidelines. The trial was registered at ClinicalTrials.gov: NCT02809287.

Inclusion criteria were as follows: (1) patients who were diagnosed with hepatocellular carcinoma (HCC) based on criteria established by the European Association for the Study of the Liver [14]; (2) single tumor with a diameter less than 50 mm; (3) tumor located in segment VI, VII, or VIII, and near the surface of the liver, without noteworthy surgical contraindications; (4) no major vessel or bile duct invasion or metastasis; (5) Child–Pugh grade A or B, or grade C that returned to grade A after liver-protecting treatment; and (6) a signed informed consent form from patients.

Exclusion criteria were as follows: (1) major vessel or bile duct invasion; (2) recurrent HCC; (3) extrahepatic metastasis; (4) Child–Pugh grade C; (5) noteworthy surgical contraindications; and (6) patient refusal to undergo laparoscopic hepatectomy.

### Observation indicators

Operation time, intraoperative blood loss, postoperative fasting duration, postoperative drainage duration, and duration of postoperative hospital stay were recorded.

### Treatments (video)

#### Operative position and trocar placement

**Fig. 1:**
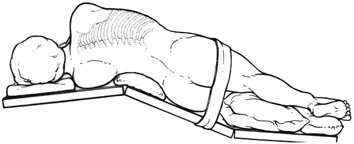
Sketch map of the left jackknife position

**Fig. 2:**
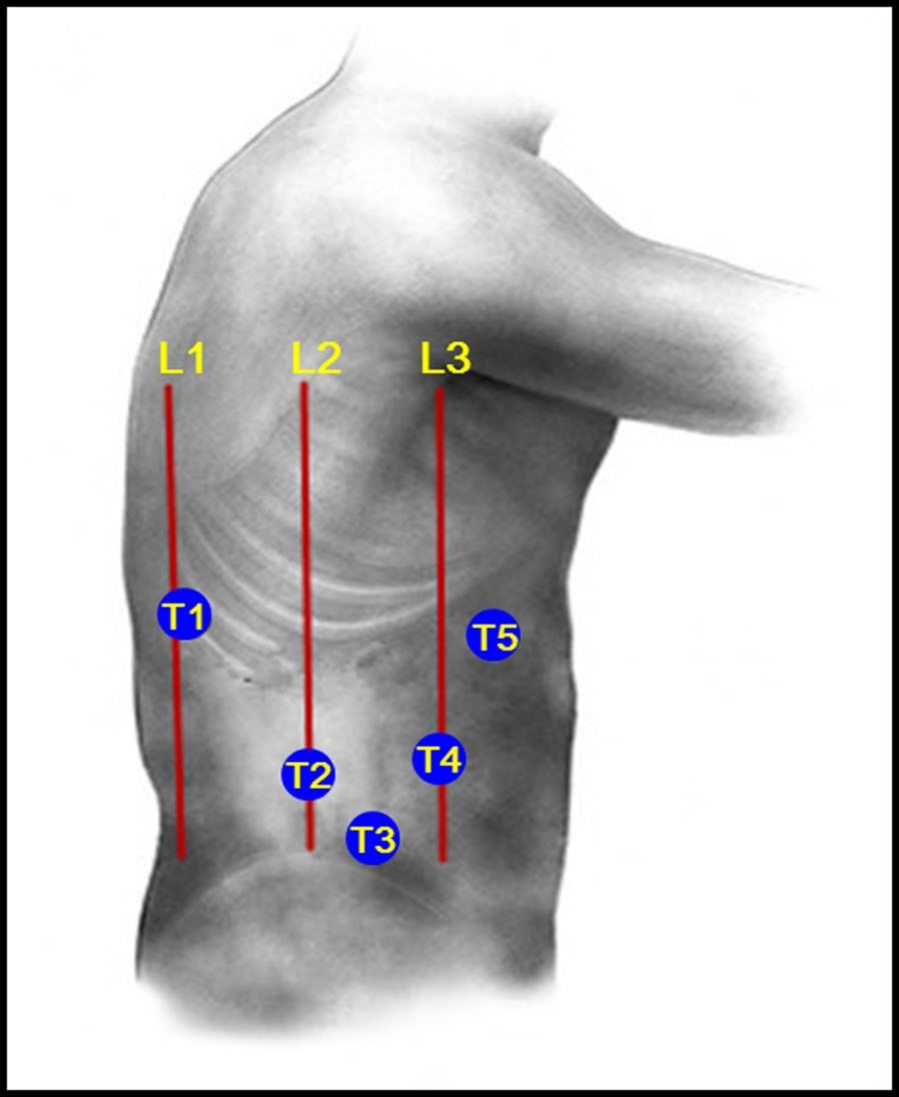
Trocar placement in the left jackknife position. *L1* posterior axillary line, *L2* midaxillary line, *L3* anterior axillary line, *T1* main operating hole 1, *T2* main operating hole 2, *T3* observation hole, *T4* auxiliary operation hole 1, *T5* auxilliary operation hole 2

Patients were first placed on their left side, then the lumbar region was elevated by adjusting the operating table to a 120° angle, presenting the lumbar region, pelvis, and lower limbs in the shape of a jackknife or “∧” (Fig. 1). A 10-mm incision was made as the main opening for observation at the junction of the umbilicus, the anterior superior iliac spine, and the anterior axillary line (T3, Fig. 2). Artificial pneumoperitoneum was established with CO_2_, with the pressure maintaining at 12–14 mmHg, before a camera was inserted for observation. Another 12-mm trocar was inserted at the intersection of the midaxillary line and umbilical level line as the main operating hole (T2, Fig. 2). The other three 5-mm trocars were located at (1) the intersection of the posterior axillary line and the eleventh rib space (T1, Fig. 2), (2) the intersection of the anterior axillary line and the umbilical line (T4, Fig. 2), and (3) the intersection of right clavicle midline and rib arch (T5, Fig. 2).

#### Surgical approach

First, the liver was mobilized by cutting the ligamentum tereshepatis, right triangular ligament, and hepatorenal ligament. Then, intraoperative ultrasonography was performed to identify the border and extent of the tumors and confirm the resection line. The Gillison sheath was dissected, and, if necessary, the right posterior portal vein was pre-ligated. Ultrasonic scalpel, electric scalpel, and hem-o-lock clips were used to sever the liver. Then, the resected surface was carefully stanched and sealed. Finally, the operating field was washed and cleaned, and the specimen was taken out.

## Results

Between January 2015 and January 2016, 10 patients underwent laparoscopic hepatectomy with the left jackknife position. All patients were men, with a median age of 52 years (range 43–70 years) and a mean body mass index of 22.4 ± 1.8 kg/m^2^. Lesions were located in segment VI (three cases), VII (four cases), or VIII (three cases), and the median tumor size was 31 mm (range 23–41 mm) in diameter.

Ten patients completed laparoscopic hepatectomy, with a mean operation time of 166 ± 38 min and a mean intraoperative blood loss of 220 ± 135 mL. The median postoperative fasting duration was 1 day (range 1–2 days); the median postoperative drainage duration was 2.5 days (range 2–3 days); and median postoperative hospital stay was 4 days (range 2–7 days). No major postoperative complications were observed.

## Discussion

As a minimally invasive surgery, laparoscopic hepatectomy has great advantages over traditional open surgery in terms of hospital stay, complications, and surgical wound healing [15, 16]. Because of poor operative exposure, small tumors located in segment VI, VII, or VIII are relatively difficult to be resected via laparoscopic local excision with the conventional supine position. Patients with these tumors are prone to undergo laparoscopic right posterior lobectomy, right anterior lobectomy, or even right hepatectomy, which may aggravate postoperative hepatic dysfunction due to the loss of more normal liver tissue. Some patients even had undergone conventional open surgery in past years.

Good exposure of the hepatic lesion is the pre-condition for a successful laparoscopic hepatectomy [17]. In this study, we employed this novel position—the left jackknife position—in laparoscopic hepatectomy, allowing gravity to pull and rotate the liver and full exposure of lesions located in the posterior lobe (including liver segment VI, VII, or VIII) and the retrohepatic tunnel. With direct and expanded surgical vision, blood loss was reduced, and a safe procedure was conducted.

Laparoscopic hepatectomy is relatively difficult for lesions located in segment VI, VII, or VIII. One reason is that a lesion in the upper part of the right lobe of the liver is close to the diaphragm, leading to a difficulty in hepatic dissociation. Another reason is that dissociation of a lesion close to the second hepatic portal may injure the major vessels. In a regular laparoscopic hepatectomy, a horizontal position is applied [18]. After dissociation of the hepatic ligaments, the liver is pulled to the left side or the upper left side to expose a lesion that is located in segment VI, VII, or VIII. However, the problem that the surgeon cannot gain direct vision during the procedures may result in increased blood loss.

Our study had several limitations. First, it had a small sample size (10 cases); more cases need to be assessed. Second, compared with the supine position, the jackknife position may cause a poor exposure of the hepatic portal area, which would make hilar dissection difficult when hepatic inflow blocking is required. Third, performing a laparoscopic hepatectomy with the patient in the left jackknife position is more time consuming than performing it when the patient is in the conventional supine position.

Our experience shows that the left jackknife position can offer favorable surgical vision for laparoscopic hepatectomy, which is useful to resect lesions located in liver segment VI, VII, or VIII. We recommend that this position be used for right posterior lobe resection in laparoscopic hepatectomy.
